# Guest Editorial: Can we really reduce error rates?

**DOI:** 10.1120/jacmp.v12i2.3626

**Published:** 2011-05-16

Medical Physics has finally made the front page of most major newspapers in the US, including *The New York Times*. Unfortunately, the headlines have highlighted some of our spectacular failures rather than spectacular achievements. After decades of flying “under the radar screen” we've finally made the headlines in a most unfortunate manner. Alas, “medical physicist properly calibrates SRS beam and doesn't kill anybody” sells fewer newspapers than “patient lies dying from radiation overdose”. But why so many recent reports of medical physics errors? Bad timing? Vagaries of chancey Or have medical physicists hit a new high (or low) in incompetence? I hate to say this, but I fear the latter. And one must ask, “How can this be?”

Haven't we (that is, collectively the medical physics community) spent the last 10–15 years toughening the entrance requirements into medical physics? We now have CAMPEP degrees and residencies, Doctorates in Medical Physics, an ABR exam roundly touted as being much tougher, licensing requirements in four of the most populous states in the country, MOC and continuing education requirements, etc. How can things be getting worse, rather than better?

There are obvious factors working against us. We are in the midst of an exponential and seemingly never‐ending increase in complexity of treatment and diagnostic techniques, with new technologies appearing every day with which even the most competent practitioner can't keep pace. As well, shrinking budgets and increased workloads have increased everyone's stress levels.

But I think the real problems runs much deeper, with at least five more serious factors at work:

1. We are training a new generation of medical physicists to be technicians and medical specialists rather than physicists and scientists. We are teaching “cookbook” medical physics instead of “thinking” medical physics.
2. We are not teaching history or responsibility. How many medical physicists feel personally responsible for the safety of their patients or truly understand that their mistakes can adversely affect the safety of another human being? How many new medical physicists even know what the “Therac‐25 disaster” was all about, or how many patients were killed and injured when a medical physicist miscalibrated (or more accurately, failed to calibrate) a Cobalt‐60 teletherapy unit 40 years ago in Ohio?
3. All of our attempts to improve training and credentialing have targeted individuals rather than institutions and processes. Even the best trained individuals – when put into a position where stress levels are too high, where policy and procedure guidelines are poorly conceived or nonexistent, where there is lack of enforcement and inadequate monitoring of performance – are going to make mistakes.
4. There are no uniform national standards for reporting errors, no national database, and no way to learn from the mistakes of others.
5. There is not a single government agency, state or federal, responsible for regulating the so‐called “practice of medicine”, or disciplining practitioners who've demonstrated an exceptional level of incompetence.

Wrong, you say. We have the NRC, FDA, and state health departments. Well, yes and no. None of them regulate the “practice of medicine”. The FDA regulates primarily manufacturers but, with the explosion of new technologies, even “experts” in the field can't keep pace with things. So how can we expect the woefully understaffed and underfunded FDA to regulate product safety? The NRC and state departments of health can only discipline people for not reporting errors after they make them, but can effectively do nothing to actually prevent errors. If you don't believe this, just look at the “slap on the wrists” the NRC recently gave to the individuals responsible for over 90 botched implants at the Philadelphia VA Hospital. And how many times has a state revoked someone's license, or the ABR revoked someone's certification for anything other than criminal activities?

So, what does the public make of this mess and our inability to police ourselves? I recently posted on the *Medical Physics* List Server a letter from the daughter of a patient who was severely overdosed in a MammoSite® treatment because of a medical physics mistake. The patient died 15 months after the accident, and the daughter then tried to find out exactly what had happened and what corrective actions had been taken. She tried contacting the hospital, state department of health, NRC, HHS, local politicians, and professional medical societies. To make a long story short, she ran into a brick wall of denials and comments such as: “not my job,” “not within my jurisdiction,” and “we've followed all proper procedures and done everything we could”.

In short, the family got the impression that nobody cared, nobody was willing to take responsibility, nobody learned anything from the event, and nothing was going to change as a result of the accident. Even worse, as I read her letter and looked further into the matter myself, I too got that impression. Nothing was going to change! Lots of talk. Lots of letters on the List Server. Nice session on patient safety at last year's AAPM meeting. Great position papers written by ASTRO and ACR. But nothing has actually changed. The hospital where the accident took place continues to do business as usual; no changes in state or federal laws protecting patients from future errors were made, and the individuals responsible either continued to work at the very same hospital or moved to another hospital where nobody knew about their involvement. If, in fact, significant sanctions were imposed and/or significant changes were made, both the hospital and the regulatory agencies did everything humanly possible to insure that neither the patient's family nor the public knew anything about it. Talk about bad public relations!

As Peter Finch (aka, Howard Beale) said in the movie *Network*, “I'm mad as hell and I'm not gonna take this anymore!” As a profession we have to do a better job of preventing errors and policing ourselves. It's about time we got “mad as hell” and did something other than talk. Here are a few suggestions.

AAPM should initiate a massive new education program. First, we need to educate our own members. We need courses and symposia on the history of errors or, as David Letterman would say, “stupid medical physics tricks”. We need a course on “physics errors” in every CAMPEP program and we need “physics error” questions on the board exam. Perhaps we should add an essay question to the Board Exam. Every candidate has to write a three‐page single‐spaced essay entitled “the history of medical physics mistakes and how I can kill as many patients as possible by not doing my job properly”. Second, we need a public education program informing the public on what medical physics is and why they shouldn't go to a hospital that doesn't have a medical physicist. We should publish brochures for patients informing them of questions they should ask their doctor before they consent to getting treated: Does your hospital have a QA committee? Do you have a policy and procedures manual? Do you have a full time medical physicist and is he/she certified, etc?

We have to educate new physicists (and also some old ones) on just what their responsibilities are and how much authority they have. Yes Virginia, you can tell an MD that this patient isn't getting treated today because I'm not finished checking the treatment plan. Yes, you can take a linac out of service if you think there's a safety problem that needs to be investigated.

We may not have much control over congress or government regulatory agencies, but we do have control over many others things. Why can't the ABR “decertify” someone who demonstrates an exceptional level of incompetence? They already decertify people for not paying their renewal fees. Why can't they decertify people who lose their competency? If the government won't start a national database for radiation accidents, let's do it ourselves. It would have to be voluntary, but it's a start and anything would be better than what we have now.

We need to re‐examine the recent, well‐intentioned changes that have been made in medical physics education. As aluded to earlier, the educational focus is now on details and narrowly focused on “medical physics” rather than physics. I fear that we are losing touch with our roots as physicists, which is the one thing about us that separates us from all other professionals in a hospital. We want to be “medical specialists” rather than physicists, as if that were something very special. But being a “medical specialist” is nothing special. Hospitals are filled with “medical specialists,” but they're not filled with physicists and, as such, we have historically provided something that really is special to the hospital skill set. Physicists (and engineers) have made modern radiation therapy and radiology possible. Physicians didn't do it, we did it. And now, by our own design, we are losing the very thing that has made us special, and I can't help but speculate that the increases we are seeing in error rates is somehow related to that. We're training technicians, not physicists or scientists.

Let's be physicists. Let's start policing ourselves, and let's stop waiting for ASTRO or ACR or RSNA or the government to fix things for us. Because that could take time and our patients can't wait any longer!

Howard I. Amols, Ph.D.

Chief, Clinical Physics Service

Memorial Sloan Kettering Cancer Center

New York, NY 10021

